# Supplementary material for: Engineering, decoding and systems-level characterization of chimpanzee cytomegalovirus
Source: PLoS Pathog. 2022 Jan 4;18(1):e1010193. doi: 10.1371/journal.ppat.1010193 (PMC8759705; doi:10.1371/journal.ppat.1010193)
Supplement: Attachment — Submitted filename: Point-by-point response.docx [file ppat.1010193.s022.docx]

**POINT-BY-POINT RESPONSE TO REVIEWERS’ COMMENTS**

**Editor summary**

As you can see from the attached reviews, every reviewer recognized your study as important to the field and clearly appreciated the work you invested into writing and preparing this manuscript. Nevertheless, while all reviewers showed excitement over the novel BAC and CCMV model system you have created, some issues were raised that need to be addressed. There are two points in particular that were brought up during the review process that I would like to highlight.

***Response:*** *We thank all reviewers for their expert advice which helped us a lot to improve our manuscript.*

First, while all reviewers recognized the importance of a CCMV BAC, the question was raised as to how far this BAC actually reflects circulating WT CCMV isolates as multiple genes were lost during the cloning process. Furthermore, the BAC additionally acquired an unusual amount of sequence differences compared to the original viral sequence, either potentially indicating genome instability or sequencing errors. Reviewer 1 also raised additional concerns arising from the sequences differences between the generated BAC and the original parental virus, hence the authors should re-evaluate their identified mutations and examine potential effect these adaptations could have on the resulting BAC derived virus for viral genes identified as possibly affected by the reviewer.

***Response:*** *We thank the reviewers for bringing up these important points. To address their concerns, we implemented several additional analyses in the manuscript, leading to new figures 2A, 4C, 4D, S7 and S8 (see the point-to-point response below).*

Secondly, both reviewer 1 and reviewer 3 noted that the experiments performed to characterize the CCMV BAC derived virus were actually performed on human cells, hence in cross-species infections. As explained in the reviews, this can be suboptimal as this could make the interpretation of the generated results more complicated as the virus is not adapted to this host species. Reviewer 3 suggested potentially useful approaches that authors could attempt to address this issue.

***Response:*** *We thank the reviewers for their very helpful suggestions. We truly regret that we can not conduct infection experiments with chimpanzee cells without causing an unreasonably long delay of the manuscript. As we outline below, due to strict rules and regulations applying to the distributions of chimp materials it is simply impossible for us to perform such experiments on short notice.*

**Part I: Reviewer summary**

**Reviewer #1**: This study generates a BAC clone of a chimp CMV strain, then characterises it genomically, as well as by transcriptomics and proteomics. Given that chimp CMV is the closest relative to human CMV, studying the two can provide novel information, and this will be greatly facilitated by having a BAC clone.

***Response:*** *We appreciate this positive comment by reviewer #1.*

**Reviewer #2**: In this lovely 'powerhouse' manuscript, the authors describe the BAC cloning and careful proteomic and transcriptome characterization of the chimpanzee cytomegalovirus (CCMV), which is the closest known animal virus relative to human cytomegalovirus (HCMV). The authors interpret their data cautiously, while also presenting a comparative analysis against data from the Weekes' laboratory. Their analyses adds enormous value to what would already be a highly appealing resource paper. HCMV is an important human pathogen that has also proven to be a source of myriad insights into molecular and cell biology (promoters, US11 / ERAD) and the immune system (ULBP's etc, NK cell activation and viral evasion thereof, et cetera). Until the work described in this study, there have not been any available reverse genetics system established to study CCMV, which is frankly shocking. Although Rhesus CMV (RhCMV) is an established primate model system, and the use of chimpanzees as model systems for biomedical research is no longer permitted, the comparative value of CCMV cannot be overstated-- especially since many RhCMV paralogs are functionally divergent from HCMV.. As the authors discuss, even certain CCMV gene products are not shared in HCMV and paralogs like UL148 exhibit only limited features in common. Not to get lost in the weeds here, having the CCMV genome cloned as a BAC now allows for careful examination of the acquisition of evolutionary novelty and divergent features, which will may in turn shed light on immune differences between primates.. as reflected by the viruses that infect them. I very rarely encounter such carefully prepared manuscripts.

My only very minor critique of the work is that on lines 224-243 they go over the temporal classes for different viral genes -- based on their expression. I never understood why Mike Weekes' otherwise excellent temporal viromics paper that established these classes for HCMV did not accurately encompass IE2-86 kd (UL122). This gene product is expressed both as an IE gene that rises and falls at very early times post infection (~4 hpi - 12 hpi) but then rises again as viral DNA synthesis commences, and keeps increasing up to a plateau that is maintained until the end of lytic infection. It's not just phenomenology because we know IE2 is critical for transactivation of E genes.. and IE2 is transiently expressed in abortive reactivation from latency.. the second phase of IE2 expression accompanies lytic progression. It may well not be appropriate for these authors to find a temporal category or class that fits this important gene.. but I thought I'd at least mention it here.

Congratulations to the authors. I am happy to sign this review.

Jeremy Kamil

***Response:*** *Dear Jeremy, thank you for this very positive feedback on our manuscript. We fully agree that the CCMV BAC will be of great value for future comparative studies on CMV gene function. We agree that UL122/IE2 expression is special in several ways. Although it fulfills all criteria of a true immediate early gene, its expression levels are kept low at early times of infection by a negative autoregulatory feedback loop, mediated by IE2 binding to the cis repression sequence (crs) in its own promoter. At late times, IE2 protein accumulates to high levels, leading to its classification as a TP3 gene. It is clear that a cluster analysis of temporal expression data can not cover all aspects of gene regulation.*

Reviewer #3: CCMV is the closest known relative of HCMV with its genome highly collinear with that of HCMV. The sequencing data of CCMV were utilized to assess the coding potential of wild-type HCMV in early 2000 by Dr. Andrew Davison. CMVs are highly adapted to their host species and cross-species infections of CMVs are extremely unlikely to happen. Therefore, in vivo examination of all aspects of CMV biology relies on animal models and their respective CMVs. The most commonly used models for CMV are mice, rats, guinea pigs, and rhesus macaques. The close evolutionary relationship of nonhuman primates to humans is mirrored in the evolutionary relationship of the primate CMV genomes to HCMV. Among them, CCMV shares the highest level of similarity in genomic structure, coding capacity, and sequence homology. However, CCMV has never been considered as a model virus for HCMV due to the fact of chimpanzee models being phased out in most areas of research.

In this manuscript entitled “Engineering, Decoding and System-Level Characterization of Chimpanzee Cytomegalovirus”, the authors cloned the CCMV genome into BAC for the first time and subsequently repair the genome to near full-length. They also conducted the functional experiment demonstrating that, by repairing the frameshift mutation in UL128, productive CCMV infection could be re-established in endothelial and epithelial cells. In the second half of the manuscript, the authors re-evaluated the CCMV coding potential by systemically analyzing the transcriptome and proteome of infected cells and identified 14 novel ORFs, splice variants, and regulatory RNAs. Further, k-means clustering analyses of viral and host gene expression indicating that viral proteins cluster into five distinct temporal classes and host response to CCMV infection are in line with known hallmarks of HCMV infection.

Overall, this manuscript is well written and all experiments shown here were well executed. Although the transcriptome and proteome analyses part of this manuscript is very descriptive, I like the authors’ attempts to incorporate the datasets from other studies in the data analyses. Having said that, I strongly feel that conducting all experiments in human fibroblasts markedly reduced the biological significance of the data shown here.

Co-evolution of CMV with their host species over millions of years has led to some unique interactions between viral proteins and host intracellular/extracellular proteins (of various cell types). In the past, primate CMVs (chimpanzee, baboon, rhesus macaques) were isolated and adapted in cell cultures using human fibroblasts due to their readily availability. Chimpanzee fibroblasts are now possible to obtain and have been shown to have unique gene expressing profiles than their human counterparts. The authors should consider including some comparative analyses of CCMV infection in chimpanzee fibroblasts. Otherwise, one may suspect the discrepancies of temporal profiles (of viral gene expression) and their impacts on host cells (at both RNA and protein levels) between HCMV and CCMV are experimental artifacts resulting from CCMV replicating in cells not from its natural host.

***Response:*** *We thank the reviewer for pointing out how our gene expression analysis of chimp CMV infected cells may benefit from comparative experiments in chimpanzee fibroblasts. To obtain chimpanzee fibroblasts, we have contacted about a dozen different laboratories and biobanks in the US and Europe. To cut a long story short, we had to learn that chimpanzee fibroblasts are NOT readily available to us for a number of reasons. So we decided to include instead a new paragraph in the manuscript discussing the importance of performing such comparative analyses in the future (lines 359-367). We hope that the reviewer is okay with that.*

**Part II – Major Issues: Key Experiments Required for Acceptance**

**Reviewer #1**:

**1.** The inability to re-insert IRS1-TRS1 is disappointing, although a well recognised risk with inserting BAC cassettes into this region, based on previous experience with HCMV BACs.

**a)** Does the genetic deletion here effect IRS, i.e. does their virus still form four genomic isoforms?

***Response:*** *This is a very valid question and we have addressed it by performing diagnostic PCRs overspanning the internal repeat region of CCMV. PCR primer pairs, matching both strain Heberling and clone Phan9 sequences, were designed for all four possible orientations of the UL/US regions. By comparing the obtained length of PCR products with the expected size and by sequencing the transitions from UL/US into internal/terminal repeat regions, we were able to confirm that CCMV BAC-Phan9 is still able to form all four genomic isoforms (****see new Fig. S7****), despite its defect in IRS1/US1. As a control, we also included the parental Heberling strain in this isoform analysis.*

**b)** Most importantly, do genes that may have transcriptional termini in this region (e.g. US2/3/6 etc) express properly? One of the reasons I ask is that US2 etc seem to express very early, then drop off almost instantly, which seems very odd for genes that play a major role in immune evasion throughout the virus lifecycle. Is this due to the lack of the IRS1-US1 region?

***Response:*** *Although we cannot rule out that the lack of IRS1/US1 influences the expression kinetics of upstream US genes, the fact that the RNA sequencing read coverage shows clear boundaries between US2, US3-US6, US7 and US8-US9 (****see new Fig. S8****) suggests that these genes are expressed as separate transcriptional units and not as polycistronic transcripts terminating in the IRS1/US1 region. We now elaborate on this observation (lines 181-183). Unfortunately, we can not easily compare the temporal profiles of US genes during BAC-Phan9 infection with the parental wild type strain, as the CCMV Heberling stock carries a deletion of the IRS1-US10 region within the majority of genomes. This defect became obvious when we compared the proteomic profiles of either Heberling or BAC-Phan9 infected cells side by side and found that CCMV Heberling was unable to properly express US2-10 genes (****see new Fig. 4C****). Importantly, this was the only major difference between both viruses. The IRS1-US10 deletion within the Heberling virus pool was further confirmed by revisiting the whole genome sequencing data at this specific locus (****new Fig. 2A****). Considering that the Heberling strain is currently the only CCMV isolate available, the finding of an unstable genomic region underlines the importance of fixing and repairing the CCMV genome as a BAC.*

**c)** Does the virus downmodulate MHC-I in the same way as the parental virus?

***Response:*** *We agree that this is an efficient way to measure the biological activity of the repaired US2-US11 region. We performed flow cytometry to analyse MHC-I surface expression of infected cells using HCMV Merlin as positive and mock infection as negative control. We found that the parental Heberling virus has almost completely lost the ability to downregulate MHC-I (****new Fig. 4D****). This finding is consistent with an IRS1-US10 genomic deletion predominating in the Heberling virus stock (****see new Fig. 2A****). In contrast, the BAC-Phan9 derived virus has regained the ability to downregulate MHC-I surface expression, although the observed effects are not as strong as with HCMV Merlin in our experimental setting. Overall, these data demonstrate that as a consequence of the US2-US13 repair, the MHC-I antagonists US2/3/6/11 are functional during BAC-Phan9 infection.*

**2.** The power of the CCMV virus as a model system for HCMV, is when the virus is used in its natural cell type. Just because a virus grows in a cell line from another species doesn’t mean it modulates it in the same way. When used in human cell lines, there’s no way of knowing whether the presence or absence of any effects is because of species specificity. Does their virus infect chimp cell lines – I believe skin fibroblasts are available commercially?

***Response:*** *We agree that for a systematic understanding of CCMV gene functions and possible limitations or adaptations in cross-species infection, it would be desirable to compare the observed effects in human cells with infection experiments in chimpanzee fibroblasts. Unfortunately, such fibroblasts are not commercially available since the Coriell Repository has stopped distributing cells from great apes several years ago, in response to rules and regulations of the Endangered Species Act (ESA). We therefore contacted a number of research labs in the US which have worked before with chimpanzee fibroblasts. Although some of them still have chimpanzee cells in stock, they are not allowed to share or send them to Europe due to material transfer agreements and/or CITES (Convention on International Trade in Endangered Species) regulations. That is the reason why we now have initiated a collaboration with Dr. Henk Niphuis from the Virology department of the Biomedical Primate Research Centre in Rijswijk (Netherlands). He is trying to obtain some tissue material from the Apenheul Primate Park (Apeldoorn, Netherlands). However, it will take at least several months before we can get hands on an established cell line and we would clearly prefer to proceed with the present manuscript without such significant delay. Instead, we would like to design the planned series of experiments in chimpanzee cells as a separate follow-up study.*

3. According to the authors, their BAC clone has over 200 de novo mutations compared to the parental strain. This seems incredibly high. A number of studies have used NGS to show that HCMV BACs are extremely stable over time. Similarly, in Line 180, their repaired BAC clone has a number of additional mutations that are not in the region that was modified. If the BAC clone is not stable, this is a major concern, and is not in line with other studies? Does it indicate a fundamental problem with genomic stability? Can they show that their BAC doesn't acquire random mutations when being propagated and modified?

***Response:*** *We thank the reviewer for pointing out this issue regarding the genomic analysis of our BAC clones. Our first analysis relied on the published CCMV Heberling genome sequence (Accession number NC_003521). As a result, we initially plotted all found sequence variations in regard to this reference genome. However, we recognize that this could be misleading as those sequence variations that originate from the parental, genetically heterogeneous CCMV stock are not highlighted in any way. Accordingly, we* ***updated Fig. 2C*** *and plotted the fraction of variations that carried over from the Heberling pool as white bars. In contrast, the colored bars are now representing only those mutations we categorized as “de novo” because they were below the detection limit in the deep sequencing data of the Heberling stock. This way it becomes more evident that across all selected clones, only a relatively small amount of mutations are indeed created de novo. With regard to the genomic analysis of our repaired BAC clone (Phan9), we recognized that our initial variation calling included regions with low coverage, namely in the terminal repeat region (****shown in Figure 4A****). Due to these repeating sequences, illumina bridge amplification is more challenging, which is reflected by a low read coverage and relatively large amount of mutations found in this region. To eliminate sequencing artefacts, we adjusted the genome analysis of our BAC clones and set the mutation frequency threshold to 95% and thus created a higher confidence in found mutations in our BAC clones.* ***Figure 4A was updated*** *accordingly and now it has become more evident that through the entire restoration cloning procedure starting from BAC-177, only 4 variations are found within the restored US region, 6 putative variations in the terminal repeat region and no other novel mutation across the entire genome of BAC-Phan9.*

**Reviewer #2**: None needed. N/A

**Reviewer #3**:

1. Results Figure 3: Panels B and C. Please include CCMV (Heberling strain and BAC-derived) infection in chimpanzee fibroblasts.

***Response:*** *We agree that this is an important issue and we would love to do the requested experiments in chimpanzee cells. However, as already pointed out in the response to point 2 of reviewer #1, chimpanzee cells are not commercially available any longer, shipping them from US research labs to Europe is strictly forbidden and all biobanks and primate centers in Europe we have asked about, have no chimpanzee fibroblasts in storage. It is therefore simply impossible for us to perform such experiments on short notice. Instead, we included a new paragraph in the manuscript discussing the importance of such comparative analyses (lines 359-367).*

2. Results Figure 6, 7, 8: If not possible to have comprehensive analyses in chimpanzee fibroblasts, the authors should conduct kinetic analyses of select CCMV genes in chimpanzee fibroblasts.

***Response:*** *We apologize but we were unable to do these analyses due to the lack of chimpanzee fibroblasts (see above).*

**Part III – Minor Issues: Editorial and Data Presentation Modifications**

**Please use this section for editorial suggestions as well as relatively minor modifications of existing data that would enhance clarity.**

**Reviewer #1**:

1. Line 140. The authors call mutations as silent/non-coding etc based on the original CCMV annotation. Is there any risk that they have missed coding mutations in ORFs that were not annotated on the original sequence?

***Response:*** *We thank the reviewer for bringing this to our attention. After reanalyzing the variations of all BAC clones in regards to the newly described ORFs, we came to the conclusion that only gene ORFL147C carries two variations (V141A and T175A) in CCMV BAC clone 169 and 185.*

2. Can the applicants provide a schematic of the genome, with all known & novel ORFs/miRNA etc. marked?

***Response****: The CCMV BAC-Phan9 sequence is now available under the NCBI nucleotide accession number MZ151943 and includes annotations of all canonical as well as newly described ORFs and regulatory RNA sequences. Downloading the sequence in the Genbank file format generates an interactive sequence map that is viewable with DNA Sequence and Cloning software packages (e.g. Snapgene Viewer, Geneious).*

3. When restoring the US region, this is not just about making the BAC useful for immunological research. This is about making it a virus genome that actually expresses the phenotype of the real CCMV virus. Furthermore, other HCMV studies have shown that when the BAC cassette is not self-excised, it leads to genome instability when virus is reconstituted. These points need making too.

***Response:*** *The reviewer is right. We now have included this important aspect (lines 169-170), referring to the study by Murrell et al. from the Stanton lab (JVI 2016: doi.org/10.1128/JVI.02858-15).*

4. Could the applicants add some general characterisation of their proteomics profiling – e.g. how many viral/host genes were detected with >1 unique peptide. What proportion of host genes were modulated by the virus?

***Response:*** *We thank the reviewer for bringing this to our attention. We now give further details on the number of quantified proteins and number of proteins modulated by the virus in our main text: “In total, we quantified* ***4,748 host proteins*** *with at least two matching peptide species (Table S5)” (lines 252-253). “Then, we determined the top 3% most up- and down-regulated host proteins at each time point (****194 proteins****) of CCMV and HCMV infection (Fig 7A)” (lines 256-258).*

5. In the discussion of temporal classes, a slightly more thorough analysis of how CCMV and HCMV compare would be useful. For example, how much overlap is there in other classes? I’m struck by how short the timeframe of expression of the TP1 & TP2 proteins is, and also by the fact that IE1 is apparently TP2 in CCMV? Is this expected? It’s not seen in HCMV?

*In response to this point, we would like to bring our supplementary* ***Figure S11*** *to the reviewer’s attention, which contains this information. We agree with the reviewer that the average cluster profiles for TP1 and TP2 have differences comparing CCMV and HCMV. While the timing of maxima are comparable, the CCMV TP1 and TP2 then more abruptly flatten out. At present, we do not have an explanation for this observation. However, we think that the congruence between the different average cluster profiles across these two datasets is remarkable, especially given that not all homologous genes products are observed in the same clusters comparing CCMV and HCMV.*

6. In the discussion, they need to discuss the fact that they are using a chimp virus in human cells, and the limitations of doing this. They also need to make the point very clearly that they don’t know how well their BAC clone represents an authentic chimp CMV, since it has been passaged so extensively in human cells.

***Response:*** *We followed the reviewer’s recommendation and included a new paragraph in the Discussion (lines 359-367) emphasizing the need of additional experimentation and data acquisition to clarify this point.*

7. Fig 7. I’m not clear why the authors just compare the highest and lowest expressed genes. Can they not do a complete comparison of all proteins at any given timepoint?

***Response:*** *The reviewer is right. This is indeed an alternative way how the data analysis could have been performed. However, we think that our approach is more robust. The reviewer may be aware that it is sometimes challenging to cross-compare datasets that were acquired by (1) different groups, (2) different biological experimental settings, (3) different mass-spectrometers, (4) different acquisition strategies (e.g. LFQ vs TMT) and (5) different bioinformatic pipelines. For example, if we performed the analysis as the reviewer suggested, we would not be certain how to properly interpret “differently regulated proteins”. Would this be due to differences in the experimental settings or direct biological effects?*

*For this reason, we aimed for a more abstract and robust comparison and performed a GO analysis with the top 3% of down- and upregulated proteins at each of the time points for both datasets. In this way we keep the number of genes included in the GO analysis constant for both datasets, allowing a cross-comparison of the functional categories modulated by infection with HCMV and CCMV.*

8. I’m assuming that the WCL2 dataset in Table S5 has had proteins that are not in the CCMV dataset removed? If so, that needs stating.

***Response:*** *We thank the reviewer for bringing this to our attention. Yes indeed, we adjusted the list of proteins from the original Nightingale et al. (2018) data set to the proteins we observed in our CCMV data. This adjustment is now stated in the description of Table S5 (****now Table S6****).*

9. In table S4/S5, I assume these are log2 values? If so, it needs stating. Similarly ‘relative’ needs to state what it’s relative to. E.g. for Table S4, I assume it’s relative to maximal expression. It also needs to state what ‘0’ means.

***Response:*** *We thank the reviewer for pointing to this missing information. For the calculation of relative data points in table S4 we used label-free protein intensities that were normalized by the LFQ algorithm as implemented in MaxQuant (see Cox, MCP). The reviewer is right that these values are log2 transformed ratios comparing different infection time points to the mock control. We added this information to the legends of Tables S4/S5 (****now S4/S6****).*

10. Discussion - self-excisable HCMV BACs have been constructed by many others prior to its application to TB40-BAC4, it would be good to cite those studies.

***Response:*** *The reviewer is of course right and we cited additional studies (Yu et al, 2002; Stanton et al, 2010) to make this point clearer (lines 314-315).*

**Reviewer #2**: See main review. This is an excellent study.

***Response:*** *We thank the reviewer for his encouraging feedback.*

**Reviewer #3:**

1. Introduction: starting line 75, references update needed for the paragraph of RhCMV. A lot of progress has been made for the complete genomic sequence of RhCMV during the past 10 years, link below is the most recent reference: <https://journals.plos.org/plospathogens/article?id=10.1371/journal.ppat.1008666>

***Response:*** *We apologize for referring to this outdated information regarding RhCMV ULb’ and other regions which have recently been repaired. We changed this paragraph as suggested and cite now the landmark study by Taher et al. describing the naturally occurring differences in the genomes of HCMV and RhCMV (lines 77-79).*
